# Ambient PM_2.5_ Exposure Up-regulates the Expression of Costimulatory Receptors on Circulating Monocytes in Diabetic Individuals

**DOI:** 10.1289/ehp.1002543

**Published:** 2010-12-17

**Authors:** Alexandra Schneider, Neil E. Alexis, David Diaz-Sanchez, Lucas M. Neas, Shirley Harder, Margaret C. Herbst, Wayne E. Cascio, John B. Buse, Annette Peters, Robert B. Devlin

**Affiliations:** 1 Institute of Epidemiology, Helmholtz Zentrum München, German Research Center for Environmental Health, Neuherberg, Germany; 2 University of North Carolina School of Medicine, Chapel Hill, North Carolina, USA; 3 Environmental Public Health Division, National Health and Environmental Effects Research Laboratory, U.S. Environmental Protection Agency, Research Triangle Park, North Carolina, USA; 4 Brody School of Medicine and East Carolina Heart Institute at East Carolina University, East Carolina University, Greenville, North Carolina, USA; 5 Focus Network Nanoparticles and Health (NanoHealth), Helmholtz Zentrum München, German Research Center for Environmental Health, Neuherberg, Germany

**Keywords:** air pollution, costimulatory receptors, diabetes, inflammation, flow cytometry, particulate matter

## Abstract

**Background:**

Exposure of humans to air pollutants such as ozone and particulate matter (PM) may result in airway and systemic inflammation and altered immune function. One putative mechanism may be through modification of cell-surface costimulatory molecules.

**Objectives:**

We examined whether changes in expression of costimulatory molecules on circulating cells are associated with ambient levels of fine PM [aerodynamic diameter ≤ 2.5 μm (PM_2.5_)] in a susceptible population of diabetic individuals.

**Methods:**

Twenty subjects were studied for 4 consecutive days. Daily measurements of PM_2.5_ and meteorologic data were acquired on the rooftop of the exam site. Circulating cell-surface markers that mediate innate immune and inflammatory responses were assessed by flow cytometry on each day. Sensitivity analysis was conducted on glutathione *S*-transferase M1 (*GSTM1*) genotype, body mass index, and glycosylated hemoglobin A1c (HbA1c) levels to determine their role as effect modifiers. Data were analyzed using random effects models adjusting for season, weekday, and meteorology.

**Results:**

We found significantly increased monocyte expression (mean fluorescent intensity) of CD80, CD40, CD86, HLA-DR, and CD23 per 10-μg/m^3^ increase in PM_2.5_ at 2- to 4-day lag times after exposure. These findings were significantly higher in obese individuals, in individuals with HbA1c > 7%, and in participants who were *GSTM1* null.

**Conclusions:**

Exposure to PM_2.5_ can enhance antigen-presenting cell phenotypes on circulating cells, which may have consequences in the development of allergic or autoimmune diseases. These effects are amplified in diabetic individuals with characteristics that are associated with insulin resistance or with oxidative stress.

Epidemiologic studies suggest that individuals with diabetes may be susceptible to the deleterious effects of exposure to PM_2.5_ (particulate matter with aerodynamic diameter ≤ 2.5 μm) (Goldberg et al. 2006; O’Neill et al. 2007; Zanobetti and Schwartz 2001, 2002). We recently found, in a panel study on adult individuals with diabetes, significant associations between altered endothelial cell function and PM_2.5_ exposure with a lag of 1–3 days (Schneider et al. 2008). Moreover, we observed that this effect was greatest among participants who had a high body mass index (BMI); who had high levels of glycosylated hemoglobin A1c (HbA1c), a marker associated with poor insulin resistance; or who had the null polymorphism for the antioxidant enzyme glutathione *S*-transferase M1 (*GSTM1*). These latter observations suggest that more severe insulin resistance and oxidative stress may be underlying features contributing to diabetic individuals’ susceptibility to PM_2.5_-associated health effects. We also observed associations between increased blood levels of interleukin-6 (IL-6) and tumor necrosis factor-α (TNFα) with ambient PM levels with a lag of 2 days, and changes in indicators of cardiac repolarization both immediately and with 4-day lags (Schneider et al. 2010).

Recent evidence from controlled inhalation challenge studies in both healthy and susceptible individuals has shown that exposure to air pollutants has the ability to modify cell-surface markers involved in activation of the innate immune response to defend the host from infection (Alexis et al. 2008, 2010). Exposure to PM as well as ozone can up-regulate the expression of airway immune cell-surface phenotypes such as the major histocompatibility complex class II molecule HLA-DR and costimulatory molecules CD80 and CD86 [for a full definition, see Supplemental Material, Table 1 (doi:10.1289/ehp.1002543)], all receptors that mediate and promote antigen presentation (Alexis et al. 2005, 2010; Lay et al. 2007). Moreover, this effect is not restricted to mucosal surfaces of the airways. Circulating inflammatory cells also have modified surface phenotypes after inhalation challenge with airborne irritants. Frampton et al. (2006) have reported modified CD11b [see Supplemental Material, Table 1 (doi:10.1289/ehp.1002543)] expression on blood monocytes and eosinophils in asthmatics exposed for 2 hr to 10 μg/m^3^ of ultrafine particulate matter (PM). Moreover, we recently reported a link between the inverse association of PM_2.5_ exposure and lung function with cell-surface marker expression on circulating neutrophils in asthmatic children (Svendsen et al. 2007). In that study, muted baseline expression of mCD14 [see Supplemental Material, Table 1 (doi:10.1289/ehp.1002543)] was associated with significantly reduced pulmonary function (forced expiratory volume in 1 sec; FEV_1_) with each interquartile-range increase in PM_2.5_ concentration. These results suggest that asthmatic children with altered innate immune responses may have decreased capacity to respond to bacterial components on inhaled PM, potentially predisposing them to greater opportunistic infection.

Unlike for asthmatics, it is currently unknown whether diabetic individuals have impaired innate immune surface phenotypes on circulating cells as a result of exposure to PM_2.5_. Therefore, we examined the effects of ambient PM_2.5_ exposure on circulating cell-surface phenotypes in adults with type 2 diabetes. We further analyzed potential modifiers of this response related to markers of insulin resistance (BMI and HbA1c) as well as a gene related to oxidative stress (*GSTM1*), which we showed earlier was associated with enhanced endothelial cell dysfunction and increased vascular inflammation in this study population (Schneider et al. 2008, 2010). We hypothesized that there would be an association between changes in ambient PM_2.5_ levels and modified expression of circulating cell-surface phenotypes that mediate innate immune activity, and that this effect would be more pronounced in individuals who were obese, had elevated HbA1c, or were *GSTM1* null.

## Materials and Methods

### Study population

Twenty volunteers 48–72 years of age with type 2 diabetes participated in the study. Inclusion and exclusion criteria were identical to our recent report and are presented in detail in that report (Schneider et al. 2008).

Each participant visited the U.S. Environmental Protection Agency’s (EPA) National Health Effects Environmental Laboratory, Environmental Public Health Division (EPHD), in Chapel Hill, North Carolina (USA), on 5 consecutive weekdays between November 2004 and December 2005. All volunteers signed a written consent form, and the study protocol was approved by the University of North Carolina Human Studies Biomedical Institutional Review Board as well as by the U.S. EPA.

### Clinical procedures

On Monday morning of each examination week, the participants completed a baseline questionnaire on individual characteristics, health status, pulmonary and cardiac symptoms, medication, and smoking history. In each of the next four mornings, participants checked into the medical station in a fasting state and without having taken their antidiabetic medication. Upon arriving at the medical station each morning, fasting glucose concentration was measured with a point-of-care glucose meter.

### Venipuncture and assays

Each morning, approximately 60 mL peripheral venous blood was taken from an antecubital site. Both serum and plasma were obtained, stored at −80°C, and used to measure markers of acute phase response and vascular inflammation (Schneider et al. 2010). HbA1c was measured with Roche Tina Quant by LabCorp. (Burlington, NC, USA) on the day the blood was drawn and was analyzed only from the first blood sample. Study participants were also genotyped for the presence or absence of the null polymorphism of *GSTM1*, a gene that belongs to the antioxidant defense family, as described previously (Schneider et al. 2010).

### Flow cytometry

**Figure 1:**
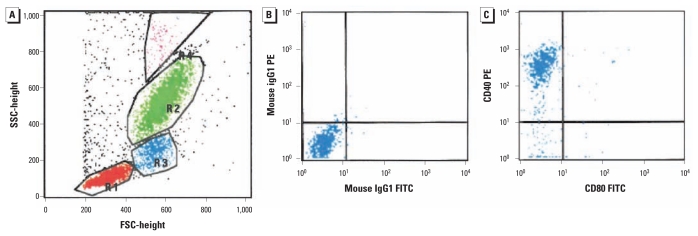
Procedure example. (*A*) Scatterplot of gated leukocyte populations based on forward (FSC) and side (SSC) light scatter properties: lymphocytes (red), monocytes (blue), neutrophils (green), and eosinophils (pink). (*B* and *C*) Quadrant plots showing monocytes in the control region using fluorescein isothiocyanate (FITC)– and phycoerythrin (PE)–labeled IgG1 as isotypic controls for CD80 versus CD40, respectively, showing > 80% CD40+ cells (*C*).

Flow cytometry was performed on a FACSORT instrument (Becton Dickinson, Franklin Lakes, NJ, USA) with approximately 1 mL fresh blood. Inflammatory cell populations (monocytes, neutrophils, eosinophils, and lymphocytes) in blood were identified and gated based on light scatter properties (forward scatter and side scatter). Whole blood was stained with saturating concentrations of monoclonal antibodies (10 μL) to cell-surface antigens (Beckman Coulter, Inc., Miami, FL, USA). After red cell lysis, inflammatory cells were resuspended in fixative (2.5% paraformaldehyde) and analyzed by flow cytometry within 24 hr using a panel of fluorescein- and phycoerythrin-conjugated monoclonal antibodies (Figure 1). The mean fluorescence intensity (MFI) of the cells stained with isotypic control antibodies was subtracted from the MFI of the cells stained with specific antibodies to get a measure of surface receptor–specific MFI. The percentage of cells that expressed a surface marker above control levels was also calculated. Surface marker expression was analyzed using the Cell Quest software (Becton Dickinson). For our analysis we analyzed only monocyte and neutrophil populations. The panel of antibodies (with associated function) we used was CD23 [low-affinity immunoglobulin (Ig) E receptor], Fcɛ receptor-1 (FcɛR1; high-affinity IgE receptor), CD80, CD86, CD40, HLA-DR (a major histocompatibility complex class II; antigen presentation), CD1a (antigen presentation), CD11b/CR3, CD54/intercellular adhesion molecule-1 (ICAM-1; complement-mediated phagocytosis, cell migration; immune complex binding), CD14, CD16, and CD64 (lipopolysaccharide; inflammation; innate immunity). For neutrophils (percentage and MFI) we analyzed only CD11b/CR3, CD14, CD16, and CD64 [for full definition of cell-surface markers, see Supplemental Material, Table 1 (doi:10.1289/ehp.1002543)].

### Air pollution monitoring

Concentrations of PM_2.5_ (average from 0900 hours to 0900 hours next day) were measured on each clinic day and for the 4 prior days with a 3000K Versatile Air Pollution Sampler (URG Corp., Chapel Hill, NC, USA) (Williams et al. 2000) located on the EPHD rooftop approximately 30 m above ground level. In addition, daily 24-hr concentrations (midnight to midnight) of fine ambient PM_2.5_ mass were obtained from a monitoring station located approximately 44 km (27 miles) east of the EPHD building and operated by the State of North Carolina. The Spearman correlation between both PM_2.5_ measurements was 0.85 across the 14-month study period, and the network data were used to impute missing rooftop data based on a linear regression model. Of 13 missing daily averages relevant for the health outcome analysis, 11 could be imputed and only two remained missing.

Continuous 2-min measurements of air temperature, barometric pressure, and relative humidity were obtained from the EPHD rooftop, and 24-hr averages were calculated.

### Statistical analyses

The study was conducted as a panel study with four repeated measurements per participant. Thus, every person acted as his or her own control, which limited the need for an adjustment for patient characteristics in the analysis.

Data were analyzed using the statistical package SAS (version 9.1; SAS Institute Inc., Cary, NC, USA). For the analysis of the PM effect, additive mixed models with a random participant effect and “compound symmetry” covariance structure were used (Fitzmaurice et al. 2004; Greven et al. 2006). Models to identify meteorologic and temporal determinants as potential confounders were built for each outcome variable separately and are described in detail in our previous report (Schneider et al. 2008). The exposure, in this case rooftop PM_2.5_ mass, was considered as an immediate (lag 0) or a delayed (lag 1 to lag 4) effect over 5 days. Effect estimates are presented as percent changes of the mean outcome variable together with 95% confidence intervals (CIs) for a 10-μg/m^3^ increment in fine ambient PM_2.5_ mass.

Sensitivity was analyzed by changing the covariance structure to first-order autocorrelation for selected PM_2.5_ results. Moreover, to assess the heterogeneity across study participants in this repeated measures study, we examined patient-specific, random slope models for selected PM_2.5_ results.

Effect modification was examined using dichotomous indicator variables that included BMI (cut-point, 30 kg/m^2^) and HbA1c (cut-point, 7%; measured only once), both characteristics associated with insulin resistance. In addition, gene–environment interaction was assessed for the null polymorphisms of *GSTM1*, a characteristic associated with susceptibility to oxidative stress.

## Results

### Subject demographics and clinical characteristics

Table 1 lists study participant demographics. All participants were current nonsmokers, and their BMIs ranged from 20 to 44 kg/m^2^; 40% (*n* = 8) were overweight (20–25 kg/m^2^), and 55% (*n* = 11) were obese (≥ 30 kg/m^2^). Forty percent of subjects had a systolic blood pressure > 140 mmHg, indicating stage 1 hypertension; most participants had a history of hypertension and dyslipidemia. Duration of type 2 diabetes ranged from 2 months to 23 years [see Supplemental Material, Table 2 (doi:10.1289/ehp.1002543)]. Most were being treated with oral antihyperglycemic medications, and roughly half were taking statins and antihypertensives. About two-thirds of the participants regularly took aspirin.

### Air pollutant and meteorology measurements

The 24-hr PM_2.5_ values over the study period were generally below the U.S. National Ambient Air Quality Standard (NAAQS) of 35 μg/m^3^, and the mean over the study period was below the annual average NAAQS of 15 μg/m^3^ [see Supplemental Material, Table 3 (doi:10.1289/ehp.1002543)]. Within the relevant 5 days of exposure (lag 0 to lag 4), three different individuals with a total of six observations experienced PM_2.5_ exposure > 35 μg/m^3^. The median of the within-individual PM_2.5_ ranges was 7.9 μg/m^3^. Correlations between PM_2.5_ and meteorology parameters as well as the time course of PM_2.5_ and air temperature during the study period can be found in a previous publication (Schneider et al. 2008).

### Description of immunologic outcomes

For a description of cell-surface marker function and mean values for the analyzed cytokines and cell-surface markers, see Supplemental Material, Tables 1 and 4, respectively (doi:10.1289/ehp.1002543). The mean values represent the average of 20 individual means (percentage and MFI), where each individual mean was the average of measurements on 4 consecutive days.

### Association of PM_2.5_ with cell-surface marker expression

**Figure 2:**
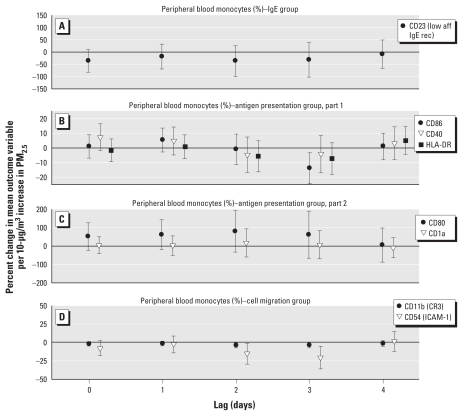
Effect estimates based on percentages of peripheral blood monocyte cellular activation markers (with 95% CIs) for immediate (lag 0) and delayed (lags 1–4) associations with PM_2.5_: IgE group (*A*), APC group parts 1 (*B*) and 2 (*C*), and cell migration group (*D*). Abbreviations: aff, affinity; rec, receptor.

**Figure 3:**
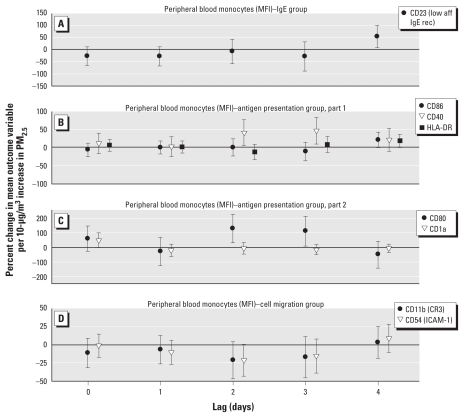
Effect estimates based on MFI for peripheral blood monocyte cellular activation markers (with 95% CIs) for (lag 0) and delayed (lags 1–4) associations with PM_2.5_: IgE group (*A*), APC group parts 1 (*B*) and 2 (*C*), and cell migration group (*D*). Abbreviations: aff, affinity; rec, receptor.

Figures 2 and 3 show results of the analysis of surface marker activation on monocytes; activation is expressed as the percent change in mean expression (based on MFI or the percentage of cells that expressed a surface marker) per 10-μg/m^3^ increase in PM_2.5_. We report significant increments of surface markers that mediate antigen presentation, that is, CD40 and CD80 at lags 2 and 3, and CD86, HLA-DR, and IgE processing, that is, CD23, at lag 4. An increase in CD1a (antigen presentation) tended toward significance at lag 0. Expression of CD54/ICAM-1, a marker of leukocyte migration, and CD11b, a receptor that mediates complement-regulated phagocytosis, had a significant decrease associated with an increment in PM_2.5_ at lag 2 and 3 (CD54 only) based on the percentage of positive cells.

**Figure 4:**
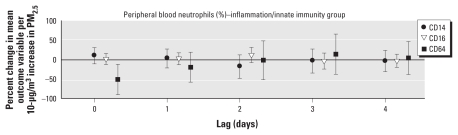
Effect estimates based on percentages of neutrophil cellular activation markers (with 95% CIs) for immediate (lag 0) and delayed (lags 1–4) associations with PM_2.5_.

**Figure 5:**
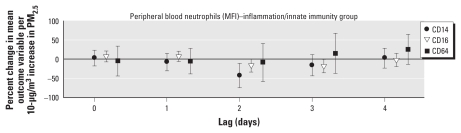
Effect estimates based on MFI for neutrophil cellular activation markers (with 95% CIs) for immediate (lag 0) and delayed (lags 1–4) associations with PM_2.5_.

Figures 4 and 5 show results of the analysis of surface marker activation on neutrophils. We observed a significant decrease in expression of CD14 (endotoxin receptor) and CD16 (receptor for IgG-mediated immune responses) based on MFI at lags 2 and 3, respectively, per increment in PM_2.5_. We found no statistically significant association with PM_2.5_ for CD14, CD16, CD64, or FcɛR1 on monocytes or CD11b on neutrophils at any time point. For absolute changes of all analyzed cell-surface markers based on an increment of 1 μg/m^3^ in PM_2.5_, see Supplemental Material, Table 5 (doi:10.1289/ehp.1002543).

### Effect modification on cell-surface markers

**Figure 6:**
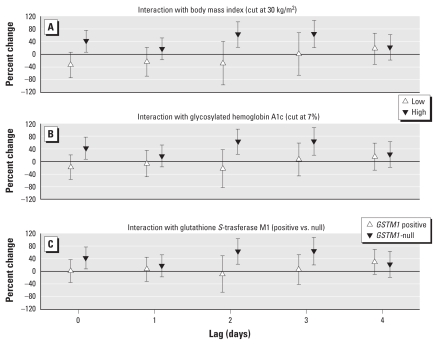
CD40 monocyte effect modification estimates (with 95% CIs) based on MFI for immediate (lag 0) and delayed (lags 1–4) associations with PM_2.5_: BMI (*A*), HbA1c (*B*), and *GSTM1* (*C*).

**Figure 7:**
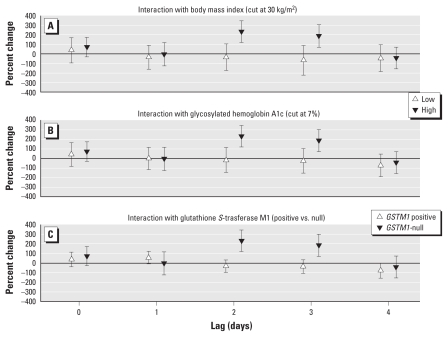
CD80 monocyte effect modification estimates (with 95% CIs) based on MFI for (lag 0) and delayed (lags 1–4) associations with PM_2.5_: BMI (*A*), HbA1c (*B*), and *GSTM1* (*C*).

For the monocyte markers CD40 and CD80, we found strong effect modification by BMI, HbA1c level, and *GSTM1*-null polymorphism based on MFI. Both markers showed far stronger associations with PM_2.5_ at lags 0, 2, and 3 in obese individuals, in individuals with elevated HbA1c, and in *GSTM1*-null individuals. The effect size in the more susceptible subgroups was comparable for both markers at lag 0, although not significant for CD80, but was much higher for CD80 than for CD40 at lags 2 and 3 (Figures 6 and 7).

### Sensitivity analyses on cell-surface markers

The estimated effects were not sensitive to changing the covariance matrix from compound symmetry to first-order autocorrelation. Random slopes showed consistent PM_2.5_ associations for almost all study participants for monocyte markers CD40 and CD80, based on MFI [see Supplemental Material, Figures 1 and 2 (doi:10.1289/ehp.1002543)]. However, for CD23 the effect at lag 4 was driven mainly by two individuals [participants 4 and 10: One was obese, both had a high level of HbA1c, and one was *GSTM1* null; see Supplemental Material, Figure 3 (doi:10.1289/ehp.1002543)]. Because the individual identification numbers order the participants in general across the study period, seasonal differences would appear as a wave pattern in these subject-specific slopes. In the absence of such a pattern, which is the case in the present analysis, we believe that we can conclude the absence of major seasonal differences.

## Discussion

Previous studies demonstrate that individuals with diabetes are susceptible to the deleterious effects of PM_2.5_ exposure (Goldberg et al. 2006; O’Neill et al. 2007; Zanobetti and Schwartz 2001, 2002). We recently reported that PM_2.5_ exposure was associated with endothelial cell dysfunction in patients with type 2 diabetes and that factors such as obesity and an elevated HbA1c level, as well as deficient antioxidant gene capability (*GSTM1* null), enhanced this deleterious association (Schneider et al. 2008). We also reported associations between PM_2.5_ exposure and changes in markers of vascular inflammation and cardiac function (Schneider et al. 2010). Here we report, for the same cohort of adult diabetic patients, that increases in PM_2.5_ exposure are associated with modified circulating cell-surface markers that mediate innate immune responses 2–4 days after exposure. Furthermore, and like our findings on endothelial cell dysfunction, this association was enhanced in obese participants, in individuals with elevated HbA1c levels, and in individuals with the *GSTM1*-null polymorphism. The cell-surface receptors that were modified were up-regulated and reflected an enhanced antigen presentation phenotype, a finding we have reported previously in controlled exposure studies on healthy individuals for other pollutants, such as ozone and endotoxin (Alexis et al. 2005, 2008; Lay et al. 2007).

Relatively few studies have reported an association between exposure to ambient PM and altered innate immune activity on circulating cells in susceptible individuals. Here we report that adults with type 2 diabetes have significantly up-regulated surface expression of CD40 and CD80 with a lag of 2 and 3 days and significantly elevated CD86, HLA-DR, and CD23 expression at a lag of 4 days on circulating monocytes. Increased expression of these surface molecules may reflect a skewing toward an antigen presentation phenotype, including IgE-mediated antigen presentation, a pathway critical in allergic asthma pathogenesis (Kim et al. 2010). We also observed a significant decrease in the proportion of monocytes that expressed CD54/ICAM-1, a cell-surface integrin involved in cell adherence and migration activity. Because several aspects of innate immunity are altered in patients with diabetes, namely, depressed polymorphonuclear leukocyte function and altered leukocyte adherence, chemotaxis, and phagocytosis (Delamaire et al. 1997; Gallacher et al. 1995; Valerius et al. 1982), these data suggest that diabetic individuals may be at further risk of innate immune dysfunction after exposure to PM_2.5_.

Among the potential consequences of monocytes demonstrating antigen-presenting cell (APC) characteristics such as up-regulated surface expression of CD40, CD80 and CD86, is the potential to activate naive T-helper cells. Although this alone may not induce a deleterious effect on adaptive immunity, it would likely cause a simultaneous decrease in the number of monocytes performing classic phagocyte functions, such as pathogen recognition, phagocytosis, and intracellular destruction of microbes. Indeed, our data showed a decrease in the proportion of monocytes expressing CD11b, the surface molecule associated with pathogen recognition and complement-mediated phagocytosis. Although significant, the absolute changes we observed in surface marker expression were relatively modest, so the interpretation of their direct impact on innate immunity remains cautious. The surface markers, however, may be useful as markers of risk and further examination.

Our data showed that certain effect modifiers significantly influenced the relationship between PM_2.5_ exposure and altered cell-surface phenotypes. These included elevated HbA1c levels, the *GSTM1*-null polymorphism, and being obese. Oxidant stress or decreased antioxidant defense capability and the resultant increase in inflammation may be a common link between these effect modifiers and perhaps offer some mechanistic insight into the effects of PM_2.5_ on type 2 diabetes. For example, obesity can itself be considered a low-grade inflammatory condition (Eklund 2009). Endogenous factors inherent in type 2 diabetes, such as oxidative stress, promote inflammation and will further affect the impact of PM_2.5_ exposure on diabetics. Evidence suggests that free radicals are formed disproportionately in diabetes by glucose oxidation, nonenzymatic glycation of proteins, and the subsequent oxidative degradation of glycated proteins (Furukawa et al. 2004). Reactive oxygen species generation is both a consequence and a promoter of inflammation. Several studies have shown that inflammation can increase insulin resistance and result in increased levels of blood sugar. Costimulatory molecules, such as CD80/86 and CD40, have long been known to be important in regulating inflammatory responses in the adaptive immune response, and more recently in innate immunity as well. Therefore, the more pronounced effects of PM_2.5_ on these cell-surface markers may reflect an enhanced underlying inflammation and a reduced ability to cope with PM-induced inflammation. *GSTM1*-null individuals have been shown to have more pronounced inflammation in response to oxidant pollutants such as diesel, tobacco smoke, and ozone (Alexis et al. 2009; Gilliland et al. 2004, 2006). In this cohort of subjects, we previously observed an association between elevated proinflammatory cytokines IL-6 and TNFα and circulating eosinophils with increments in PM_2.5_ exposure at lags similar to those reported here for cell-surface markers that mediate inflammation and IgE allergic responses, respectively [see Supplemental Material, Figure 4 (doi:10.1289/ehp.1002543)] (Schneider et al. 2010). All Pearson correlations between the cell-surface markers and the two inflammatory markers were very low, except for two that were somewhat moderate: IL-6 and CD1a (monocytes, MFI), *r* = 0.56; IL-6 and CD80 (monocytes, MFI), *r* = 0.41 [see Supplemental Material, Tables 6–9 (doi:10.1289/ehp.1002543)]. Because dendritic cells express high levels of CD1a and CD80 and produce high levels of cytokines such as IL-6, it is possible that PM_2.5_-induced skewing of cells toward an APC phenotype may in part explain these associations.

### Limitations

In this study we used daily PM_2.5_ mass values from the rooftop of the U.S. EPA EPHD building. In addition, we obtained PM_2.5_ values from a state monitoring station 27 miles away from the EPHD building. We found a strong correlation between these two sets of measurements, suggesting that we were accurately assessing ambient exposure of the study participants. However, our study shows the general limitation of accurately measuring exposure of participants, in common with all panel studies with a similar design. All the subjects lived within a 30-mile radius of Chapel Hill. Of course, there could be variable exposure to traffic as the participants commuted to work and to the EPHD building, which is difficult to capture. Although ambient exposures are measured with some imprecision, these exposure measurement errors are nondifferential with respect to either true ambient exposure or the clinical measurements. As such, these Berkson measurement errors will tend to bias our results toward the null.

A small exposure gradient within the repeated measurements of each individual only limits the statistical power of the study to detect weak associations, but none would produce a substantial bias away from the null hypothesis.

As with any study that examines many different end points, multiple testing and as a consequence the potential for false-positive results can be a problem, especially with so many effect modifiers.

## Conclusion

Similar to our previous finding in this cohort of individuals with type 2 diabetes where endothelial dysfunction was associated with increases in PM_2.5_ exposure, we found that incremental increases in PM_2.5_ exposure are associated with up-regulated blood monocyte surface expression of costimulatory molecules that promote an APC phenotype. This effect was enhanced in diabetics who had elevated HbA1c levels, had the *GSTM1*-null phenotype, or were obese. These data suggest that oxidative stress as well as severe insulin resistance may be an underlying feature contributing to the effects of PM_2.5_ exposure on innate immune responses in individuals with type 2 diabetes.

## Figures and Tables

**Table 1:** Study population characteristics: current nonsmoking subjects with type 2 diabetes mellitus (*n* = 20).

Characteristic	Mean or total *n*
Mean age (years)	60
Male sex (*n*)	12
Ethnicity (*n*)	
Caucasian	13
African American	6
Hispanic American	1
BMI (kg/m^2^)	
Mean	33
≥ 30 kg/m^2^ (*n*)	11
Mean systolic blood pressure ≥ 140 mmHg (*n*)	8
HbA1c ≥ 7%^a^ (*n*)	9^b^
Smoking (*n*)	
Never-smoker	11
Ex-smoker	9
Null polymorphism of *GSTM1* (*n*)	9^c^

a HbA1c was measured once at the baseline visit.

b Data were missing from two subjects.

c Three subjects declined permission for genetic testing.
